# Supplementary material for: Localisation of nitrate-reducing and highly abundant microbial communities in the oral cavity
Source: PLoS One. 2023 Dec 21;18(12):e0295058. doi: 10.1371/journal.pone.0295058 (PMC10735016; doi:10.1371/journal.pone.0295058)
Supplement: Attachment — Submitted filename: Rebuttal Letter.docx [file pone.0295058.s002.docx]

Artak Heboyan, Ph.D.
Academic Editor
PLOS ONE

**Rebuttal Letter for Manuscript ID: PONE-D-23-17952**

**Localisation of nitrate-reducing and highly abundant microbial communities in the oral cavity**

Dear Dr Heboyan,

I hope this letter finds you well. I would like to express my gratitude for the careful review of our manuscript titled "Localisation of nitrate-reducing and highly abundant microbial communities in the oral cavity," submitted to PLOS ONE. We appreciate the time and effort that both the reviewers and the editorial team invested in evaluating our work. Your feedback has been invaluable in improving the quality of our research, and we are eager to address the comments and concerns raised during the peer review process.

In this letter, we provide detailed responses to each of the reviewers' comments, along with a point-by-point revision of the manuscript to address the issues identified. We also include the revised version of the manuscript with track changes to highlight the modifications.

Sincerely,

Joanna L’Heureux, Ph.D., on behalf of all authors

Journal Requirements:

When submitting your revision, we need you to address these additional requirements.

1. Please ensure that your manuscript meets PLOS ONE's style requirements, including those for file naming.

RESPONSE: Thank you for providing the style requirements. We have revised the manuscript accordingly.

2. In your Data Availability statement, you have not specified where the minimal data set underlying the results described in your manuscript can be found. PLOS defines a study's minimal data set as the underlying data used to reach the conclusions drawn in the manuscript and any additional data required to replicate the reported study findings in their entirety. All PLOS journals require that the minimal data set be made fully available. For more information about our data policy, please see <http://journals.plos.org/plosone/s/data-availability>.

RESPONSE: The data are currently publicly available in the Human Microbiome Project repository that is referenced in the manuscript. The data will be available with a DOI provided through the University of Exeter ORE once the article has been accepted for publication.

Reviewers' comments:

Reviewer's Responses to Questions

**Comments to the Author**

1. Is the manuscript technically sound, and do the data support the conclusions?

The manuscript must describe a technically sound piece of scientific research with data that supports the conclusions. Experiments must have been conducted rigorously, with appropriate controls, replication, and sample sizes. The conclusions must be drawn appropriately based on the data presented.

Reviewer #1: Yes

Reviewer #2: Yes

Reviewer #3: No

2. Has the statistical analysis been performed appropriately and rigorously?

Reviewer #1: Yes

Reviewer #2: Yes

Reviewer #3: No

5. Review Comments to the Author

Reviewer #1: The manuscript describes the localization of nitrate-reducing and microbial communities in the oral sites such as attached gingiva, buccal mucosa, hard palate, saliva, supra- & subgingival plaque, and tongue dorsum. The contents (data) of the manuscript seem suitable for the publication of the PLoS One. The specific points are as follows;

RESPONSE: We thank the reviewer for helpful comments that have improved the manuscripts.

[Suggestions]
>> "Resuls" and Figure 3:
(P. 14, L. 236-237)
"Haemophilus sp. and Streptococcus sp. were found in high percentage median relative abundance in all oral sites."
The referee feels that "Haemophilus sp. in the supragingival plaque" and "Streptococcus sp.in the tongue dorsum" were not so high in the Figure 3.

RESPONSE: Lines 236-237 have been revised accordingly (‘*Haemophilus* sp. and *Streptococcus* sp. were found in all oral sites’).

>> "Discussiom"
(P. 17, L. 301-305)
"Furthermore, it is important to note that variation between samples of the same site may also occur due to differences in sampling techniques, for example, the pressure used to sample bacteria caught within the tongue crypt. Further understanding of how sampling techniques could influence the recording of bacterial abundance may be useful for future studies."

For the authors;
The referee has an experience of reading literature as follows,
1) Kikutani et al: A novel rapid oral bacteria detection apparatus for effective oral care to prevent pneumonia. Gerodontology 2012; 29(2): e560-e565), it says "The collection pressure was about 20 g, and a 1-cm distance was rubbed back and forth three times with a swab." And additionally,
2) Sato-Suzuki et al: Nitrite-producing oral microbiome in adults and children. Sci Rep 10: 16652 (11 pages), 2020.

RESPONSE: Thank you for drawing our attention to these articles that support our statement. We have revised referencing accordingly.

Reviewer #2: I have reviewed the manuscript entitled “Localisation of nitrate-reducing and highly abundant microbial communities in the oral cavity” submitted for possible publication in the journal “PLOS ONE”. The authors have done great efforts in compiling the results and describing the material and methods used in their study. The topic of manuscript is of interest of the reader and belongs to one of the most emerging issues in healthcare systems. The paper can significantly contribute to the field. As the authors have described very well, I didn’t notice any major flaw or suggestions for further improvement in the manuscript. Although some minor changes have to be done before proceeding it further rocedure. My specific comments are:

RESPONSE: We thank the reviewer for a constructive and positive review of our submission.

1. I suggest to write full forms for NO etc at it’s first appearance in the abstract and then at introduction.

RESPONSE: This has been corrected in the abstract and introduction.

2. Line 16-17: The sentence needs to be rephrased.

RESPONSE: This sentence now reads “NO3--reducing oral bacteria reduce inorganic dietary NO3- to nitrite (NO2-) via the NO3--NO2--NO pathway.”

3. Abstract: Write down the aims of study.

RESPONSE: We have revised the abstract to include the study aim (“The aim of this study was to assess whether other areas in the mouth could contain a physiologically more relevant abundance of NO3- reducing bacteria, which may be important for sampling in clinical studies.”)

4. Line 55: change as “The Human Microbiome Project (HMP) under National Institute of Health (NIH)

RESPONSE: This has been revised as suggested.

5. Line 63-68: These could be better representative for the discussion section.

RESPONSE: Thank you for this suggestion. However, we feel that this section forms a key part of the study rationale and ask the reviewer’s discretion to maintain it in the introduction.

6. Introduction: At the end of this section, mention about the study rationale and objectives.

RESPONSE: We have revised lines 80-85 accordingly. (“The aim of this study was, therefore, to determine the site(s) in the mouth where the highest abundance of NO3--reducing bacteria reside in a larger human population, such as those found in the HMP, in order to inform methodology for future studies investigating the relationships between oral NO3--reducing bacteria and human health.”)

7. Line 88: change “NIH Human Microbiome Project” to NIH’s HMP”.

RESPONSE: We do not feel that the use of genitive case is necessary here and ask the reviewer’s discretion in maintaining the commonly used terminology (e.g. https://www.ncbi.nlm.nih.gov/pmc/articles/PMC2792171/).

8. Line 137-144: Mention here about the p value, what p value was considered as significant? what parameter they used to calculate the p value.

RESPONSE: We have clarified that *P*<0.05 was considered statistically significant in line 147.

9. Line 188: The authors are suggested to write “spp.” Instead of “sp.”

RESPONSE: Thank you. We have remover “sp.” from this narrative given that only genus level data were presented for analysis.

10. Line 239 and others: remove space between number and %.

RESPONSE: This has been revised throughout the manuscript.

Reviewer #3: In the present study, L'Heureux et. al. and colleagues investigated the localisation of nitrate-reducing and highly abundant microbial communities in the oral cavity. The topic explored in this article is interesting, however, there are several points in this manuscript that need to be addressed in more detail and are listed below.

1.The authors did not provide clinical information on the study participants, e.g. healthy/disease status. Several systemic diseases are known to affect the oral microbiome and lead to dysbiosis. Please discuss this point in more details

RESPONSE: The HMP participants were healthy 18- to 40-yr-old adults, who were screened using exclusion criteria based on health history, including the presence of systemic diseases (e.g., hypertension, cancer, or immunodeficiency or autoimmune disorders), use of potential immunomodulators, and recent use of antibiotics or probiotics. We have added this information in the methods section (line 96-100).

2. The authors mention several well-established NO3-reducing bacteria, but not all reduce NO3 with the same efficiency. Please provide more information about the bacterial taxa with the highest NO3 reducing power and rank the taxa by their efficiency from top to bottom.

RESPONSE: We thank the reviewer for raising this consideration and we have added reference to previous research (Doel et al. 2005, Hyde et al. 2014) that has assessed efficiency of given species for nitrate reduction in vitro (lines 74-81).

3.Please add a new paragraph explaining the limitations of the study in more detail.

RESPONSE: We have revised the paragraph highlighting study limitations as suggested (line 305-320).

4.In Figure 2, please italicize all bacterial names.

RESPONSE: This genera names have been italicized.

5. Did the authors examine the differences between males and females for each sample? Please create a new MDS figure showing the sex differences within the samples.

RESPONSE: We checked for sex differences within each oral site before proceeding with further analysis. There were no sex differences, therefore, the male and female samples were grouped together. This information has been added in lines 130-134.

6. PLOS authors have the option to publish the peer review history of their article ([what does this mean?](https://eur03.safelinks.protection.outlook.com/?url=https%3A%2F%2Fjournals.plos.org%2Fplosone%2Fs%2Feditorial-and-peer-review-process%23loc-peer-review-history&data=05%7C01%7CA.Vanhatalo%40exeter.ac.uk%7C7e290e1aa6c64af22c1d08dbb083ace2%7C912a5d77fb984eeeaf321334d8f04a53%7C0%7C0%7C638297851954668759%7CUnknown%7CTWFpbGZsb3d8eyJWIjoiMC4wLjAwMDAiLCJQIjoiV2luMzIiLCJBTiI6Ik1haWwiLCJXVCI6Mn0%3D%7C3000%7C%7C%7C&sdata=hktp8%2Fr9xfZFcx5%2B3nOPC1tHpHd%2BBy957jBI1b%2BJKiM%3D&reserved=0)). If published, this will include your full peer review and any attached files.

If you choose “no”, your identity will remain anonymous but your review may still be made public.

**Do you want your identity to be public for this peer review?** For information about this choice, including consent withdrawal, please see our [Privacy Policy](https://eur03.safelinks.protection.outlook.com/?url=https%3A%2F%2Fwww.plos.org%2Fprivacy-policy&data=05%7C01%7CA.Vanhatalo%40exeter.ac.uk%7C7e290e1aa6c64af22c1d08dbb083ace2%7C912a5d77fb984eeeaf321334d8f04a53%7C0%7C0%7C638297851954668759%7CUnknown%7CTWFpbGZsb3d8eyJWIjoiMC4wLjAwMDAiLCJQIjoiV2luMzIiLCJBTiI6Ik1haWwiLCJXVCI6Mn0%3D%7C3000%7C%7C%7C&sdata=aDHsutL3AM3pyIlXSEwk6FX6Wf3uobwF5MFBeG0V8NE%3D&reserved=0).

Reviewer #1: No

Reviewer #2: No

Reviewer #3: **Yes:** Mohamed Abdelbary
